# Clinical efficacy of mandibular complete dentures with a resilient liner: study protocol for a multicenter randomized controlled trial

**DOI:** 10.1186/s13063-022-06657-3

**Published:** 2022-09-02

**Authors:** Katsuhiko Kimoto, Suguru Kimoto, Noriyuki Hoshi, Yusuke Sato, Yoshikazu Yoneyama, Jun Takebe, Tetsuo Ichikawa, Hiroshi Murata, Masahiro Nishimura, Shunsuke Minakuchi, Yasuhiko Kawai

**Affiliations:** 1 Administration Office of the Denture Care Society, Department of Removable Prosthodontics, Tsurumi University School of Dental Medicine, 2-1-3 Tsurumi, Tsurumi-ku, Yokohama, Kanagawa 230-8501 Japan; 2 Department of Fixed Prosthodontics, Kanagawa Dental University, 82 Inaoka-cho, Yokosuka, Kanagawa 238-8580 Japan; 3 Department of Gerodontology and Home Care Dentistry School of Dentistry, Aichi Gakuin University, 2-11 Suemoritouri Chikusaku, Nagoya, Aichi 464-8651 Japan; 4 Gerodontology and Oral Rehabilitation, Graduate School of Medical and Dental Sciences, Tokyo Medical and Dental University, 1-5-45 Yushima, Bunkyo, Tokyo, 113-8549 Japan; 5 Department of Removable Prosthodontics, Tsurumi University School of Dental Medicine, 2-1-3 Tsurumi, Tsurumi-ku, Yokohama, Kanagawa 230-8501 Japan; 6 Department of Removable Prosthodontics, School of Dentistry, Aichi Gakuin University, 2-11 Suemori-dori, Chikusa-ku, Naogya, Aichi 464-8651 Japan; 7 Department of Prosthodontics and Oral Rehabilitaion, Graduate School of Biomedical Sciences, Tokushima University, 3-18-15 Kuramoto, Tokushima, 770-8504 Japan; 8 Department of Prosthetic Dentistry, Graduate School of Biomedical Sciences, Nagasaki University, 1-7-1 Sakamoto, Nagasaki, 852-8588 Japan; 9 Department of Oral and Maxillofacial Prosthodontics, Kagoshima University Graduate School of Medical and Dental Sciences, 8-35-1, Sakuragaoka, Kagoshima, 890-8544 Japan; 10 Department of Removable Prosthodontics and Geriatric Oral Health, Nihon University School of Dentistry at Matsudo, 2-870-1 Sakaecho-nishi, Matsudo, Chiba, 271-8587 Japan

**Keywords:** Randomized controlled trial, Complete denture, Edentulism, Resilient liner, Reline, Patients’ general satisfaction, Masticatory performance, Oral health-related quality of life

## Abstract

**Background:**

During restoration of poorly fitting complete dentures (CDs) in edentulous patients, liners are used to reconstruct the concave surfaces of CDs with a new base material. These relining materials are classified into resilient liners (RLs) and non-resilient liners (NRLs), but the clinical effects of these liners and their selection criteria remain unclear. The purpose of this study is to evaluate the clinical efficacy of relining mandibular CDs using RL and NRL and to conduct a follow-up study.

**Methods:**

The study is currently being conducted at eight centers, and a parallel-group randomized controlled trial (RCT) is underway. One hundred thirty-two edentulous patients with poorly fitting mandibular CDs will be assigned to two groups based on whether they will receive RL or NRL. Participants will have an RL or NRL applied for relining their CDs using an indirect method of dynamic impressions. Data will be recorded at 1 week and 3, 6, and 12 months after denture delivery. The primary outcome will be assessment of the patients’ general satisfaction by using a 100-mm visual analog scale (VAS). Secondary outcomes will be measured as patient-reported outcomes, including food intake status and oral hygiene-related quality of life. Masticatory performance and the number of sore spots on the oral mucosa will also be recorded. Comparisons between the two groups and within-subject comparisons of pre- and post-intervention measurements will be conducted.

**Discussion:**

For dentists and prosthetic researchers in Japan, this RCT will provide information on the clinical efficacy of RL materials in comparison to RNL in CD wearers. The new evidence regarding the use of RL materials in an aging population will also be useful to dentists in other countries in their routine clinical practice.

**Trial registration:**

This clinical trial has been registered at the University Hospital Medical Information Network (UMIN) Center (UMIN000041950).

## Background

The aging of the global population, especially in developed countries, has led to an increase in the number of edentulous patients [1, 2]. In dental treatment, complete dentures (CDs) are generally the first choice to restore routine masticatory function in edentulous patients. However, due to the significant alveolar bone resorption and mucosal thinning caused by a range of oral diseases in the elderly, in clinical practice, mandibular CDs cannot be used satisfactorily in many cases owing to movement and pain. Recently, implant-supported overdentures have been considered as one of the options for maintaining and stabilizing dentures [3, 4], but due to systemic diseases, inability to visit the hospital, and cost issues, most patients often choose to repair their existing CDs [5]. The reline method is generally used for repairing a non-retentive CD. According to the Glossary of Prosthodontic Terms-9 [6], relining is defined as a procedure of resurfacing the concave surface of a CD with a new base material to precisely fit the denture base. Relining materials are classified into resilient liners (RLs) and non-resilient liners (NRLs) according to their chemical structure.

Randomized clinical trials (RCTs) have been conducted to investigate the clinical effects of newly fabricated CDs with RL materials in edentulous patients. These trials reported that the cushioning effect reduced pain and pressure ulcers during occlusion, improved patient satisfaction and masticatory performance, and reduced the number of denture adjustments [7–13]. However, no studies have investigated the superiority of CDs with RL materials over conventional CDs with NRL materials in cases of poorly fitting CDs. Therefore, we plan to conduct an RCT including the following centers throughout Japan: Tokyo Medical and Dental University, Nihon University School of Dentistry at Matsudo, Tsurumi University, Kanagawa Dental University, Aichi Gakuin University, Tokushima University, Nagasaki University, and Kagoshima University. This eight-center RCT has external validity and has been named the “mandibular CDs with resilient liner trial II” (MCORT II). The purpose of MCORT II is to evaluate patients’ general satisfaction with the relining of mandibular CDs using an RL material versus the relining of mandibular CDs using an NRL material, and to conduct a follow-up study.

## Methods/design

### Trial design

This trial is being organized by the Japanese Society for Denture Care. The trial has been developed by KK, the sponsor of the study, and conducted at eight centers (university hospitals). The trial is designed as a multicenter randomized parallel trial comparing patient satisfaction with mandibular CDs using RLs and that with mandibular CDs using NRLs, with a superiority test as the framework of this trial. Several coordinators are in close contact with the investigators at each university. Each university has an investigator and a dentist as the operator. The coordinator randomly assigns and manages the schedules of participants and investigators. The investigator obtains documented informed consent from the potential participant. They are then trained to evaluate the results of the clinical trial before measuring the data. The dentists who provided the denture treatment are not allowed to be investigators for this trial. This trial has been registered with the University Hospital Medical Information Network (UMIN) Center (UMIN000041950) on 10 October 2020.

### Organization

The coordinating center (TCC), data monitoring committee (DMC), and the steering committee (TSC) have been established for this clinical trial.

TCC consists of the chief coordinator (NH) and the coordinators (SK, YS), who are in constant contact with each other. The role of the center is to allocate patients and study design, to oversee the collection, management, analysis, and interpretation of data, and to submit the results to the steering committee.

The DMC consists of dentists who are not involved directly in this clinical trial as well as experts other than dentists. The role of the DMC is to conduct an audit of progress (report submission) to the coordinating center every 2 months and report the results to the steering committee.

TSC consists of the coordinator, the investigators and dentists from each university, and members of the DMC. The role of this committee is to audit the progress of the trial, report adverse events, deliberate and decide on important changes to the study protocol including study discontinuation, and have final authority over the trial. TSC is scheduled to meet four times a year. In addition, the contents of TSC meetings are always reported to the sponsor, and the sponsor is obligated to report annually to the funder (the Japan Society for the Promotion of Science) on progress and results to date.

### Participants

The trial will be performed at the dental hospitals of eight universities in Japan. The recruitment and treatment protocols of the present trial have been approved by the ethical review committee of the following universities: Tokyo Medical and Dental University (Register No. D2020-058), Nihon University School of Dentistry at Matsudo (Register No. EC2-042), Tsurumi University (Register No. 1840), Kanagawa Dental University (Register No. 733), Aichi Gakuin University (Register No. 612), Tokushima University (Register No. 3855), Nagasaki University (Register No. 21041910), and Kagoshima University (Register No. 200313).

The inclusion criteria are as follows: (1) edentulous patients who wanted to have their mandibular CDs repaired and (2) patients who provided written informed consent for this study. The exclusion criteria are as follows: (1) patients with physical or mental problems, (2) patients who cannot hear or read Japanese, (3) patients with dietary restrictions, and (4) patients with oral dyskinesia.

### Sample size calculation

The general satisfaction of patients will be measured as the 100-mm visual analog scale (VAS) score after 3 months of relining. The use of an RL material will be judged to be clinically effective when the VAS score of patients having CDs with RL is 15 mm higher than that of patients having CDs with NRL. Thus, the difference between the means of the two groups is set to 15 mm. The sample size is calculated using *α* = 0.05 and *β* = 0.8, based on the standard deviation of the overall satisfaction obtained in a previous study (CDs with RL: 26.3, CDs with NRL: 31.4) [7]. However, assuming that 10% of the participants would drop out, the target number of participants has been set at 132.

### Randomization and masking

**Fig. 1:**
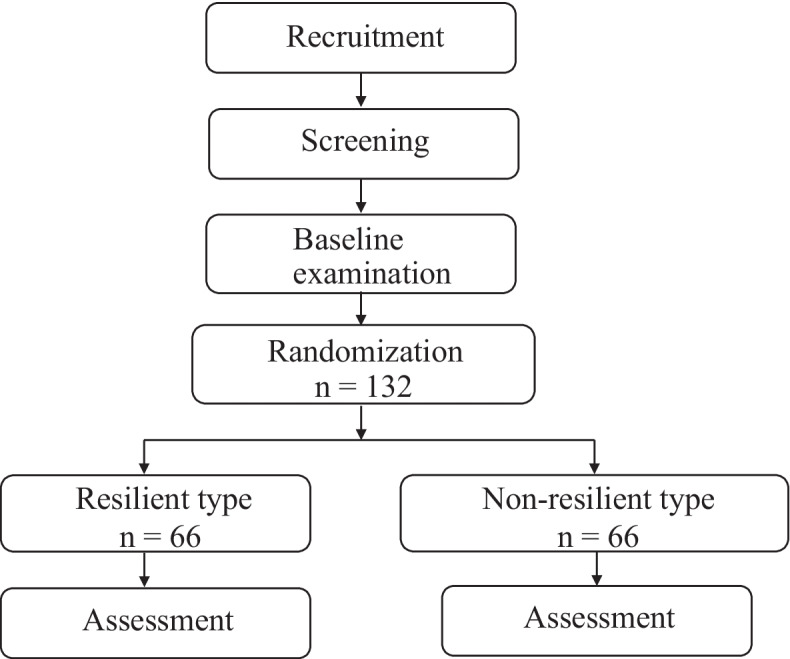
Flow diagram showing the protocol of interventions

Stratification is performed at each university hospital (Fig. 1). The assignment is performed by a block randomization method with a block size of 2. For this purpose, random numbers from 1 to 6 representing combinations of two RLs and two NRLs: 1 (NRL, NRL, RL, RL), 2 (NRL, RL, NRL, RL), 3 (NRL, RL, RL, NRL), 4 (RL, NRL, NRL, RL), 5 (RL, NRL, RL, NRL), and 6 (RL, RL, NRL, NRL) are generated in Excel (Microsoft, USA) to create an allocation table. Kanagawa Dental University will centrally manage the allocation list for each university, and the investigators of each university will contact the steering committee by e-mail with each patient’s registration details to determine the intervention method. While both the dentists and patients in this trial will not be blinded to the intervention, they will be blinded to the outcome measures and analysis.

### Intervention

The intervention for this trial will be the application of an RL material (Reline 2 Extra Soft; GC, Tokyo, Japan) or an NRL material (Rebase III [Normal]; Tokuyama Dental, Tokyo, Japan) to the mandibular CD. This trial uses an indirect lining method with the application of dynamic impression. Tissue conditioner (Tissue Conditioner II Soft; Shofu, Kyoto, Japan; Tissue Conditioner; GC, Tokyo, Japan) will be used for recording the dynamic impression. All prosthetic procedures will be performed by a prosthodontist with at least 5 years of experience. In principle, the indirect method should be performed by a dental technician. The technique used is as follows:

1. A dynamic impression is made using a tissue conditioner on the CD to be indirectly relined. The dynamic impression should be recorded when the patient does not complain of pain during mastication. Considering the surface properties of the tissue conditioner, an interval of approximately one week between uses is appropriate.
2. A super-hard plaster (New Fuji Rock; GC, Tokyo, Japan) is poured on the dynamic impression surface to make a plaster model, which is flasked for denture processing.
3. The flask is opened, the tissue conditioner is peeled off from the mucosal surface of the model, and the mucosal surface of the denture base is trimmed to create space for the reline layer. The thickness of the reline layer is approximately 2 mm.
4. After applying the adhesive material according to the manufacturer's instructions, the CD is lined with the relining material (RL or NRL), cured, morphed, and polished. The relined CD is then delivered to the patient. Post-denture delivery follow-up appointments will be scheduled, and any necessary adjustments will be carried out.

### Outcomes

The basic patient information recorded will include age, sex, years of denture use, and number of dentures fabricated. In addition, treatment difficulty index measurements [14, 15] and evaluation of used dentures [16] will be performed. Since patient satisfaction with CDs is a clinically important factor for evaluating the therapeutic effect of CDs with RL, the primary endpoint will be patients’ overall satisfaction with the CDs, which will be determined using a 100-mm VAS [17]. Patient satisfaction will be assessed comprehensively using a questionnaire containing eight assessment items: general satisfaction, ease of speaking, ease of cleaning, retention, stability, comfort, and esthetics. Participants will be asked to rate their satisfaction with each item in the questionnaire by using a 100-mm VAS to evaluate their current CDs. Data will be recorded as continuous variables at 1-mm intervals by using calipers from the left end. Overall patient satisfaction will be measured four times: at baseline, 7 days, 3 months, 6 months, and 1 year.

**Fig. 2:**
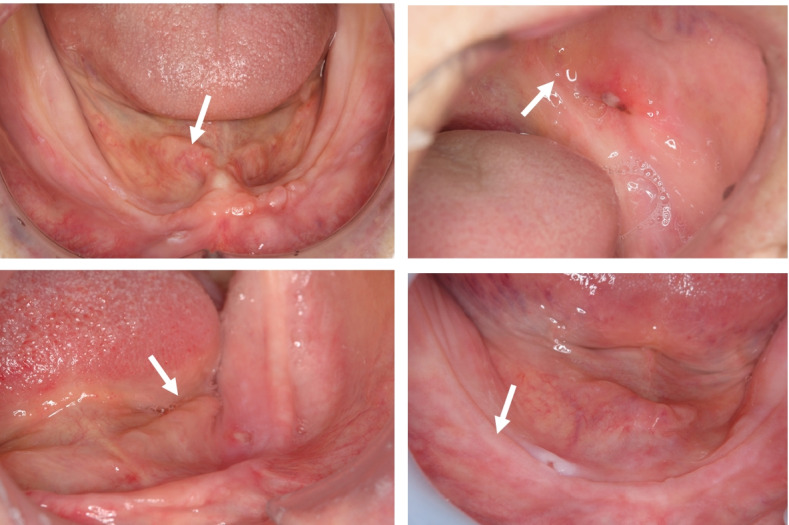
Photographs of pressure ulcers caused by representative dentures used for investigator calibration

In addition, to comprehensively understand the clinical effect of CDs with RL material, the Oral Health Impact Profile for edentulous subjects (OHIP-EDENT) [18], masticatory ability [19, 20], food intake [21, 22], and the location and number of mucosal pressure ulcers will be examined as secondary outcomes. Photographs of mucosal pressure ulcers will be shared among investigators to adequately calibrate assessments of the mucosal surface (Fig. 2).

### Trial schedule

The trial schedule is outlined in Table 1.

**Table 1:**
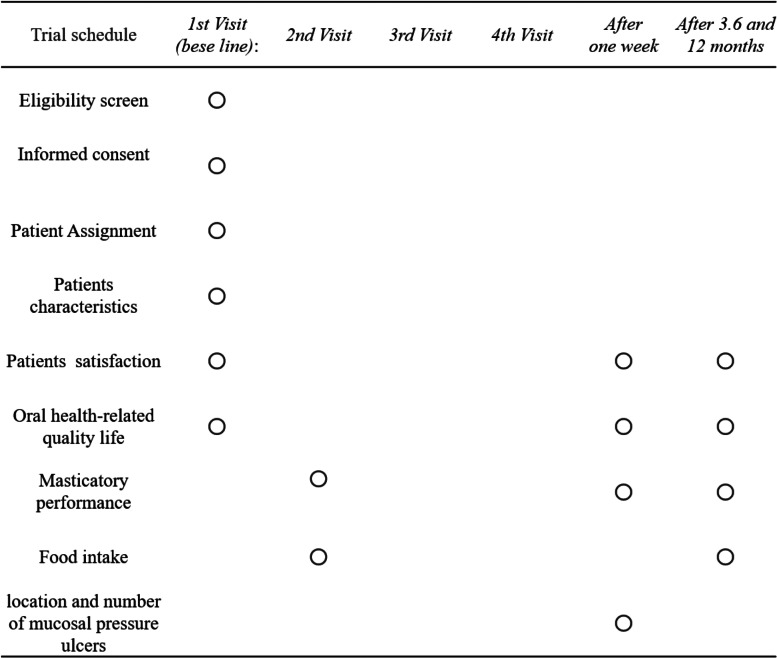
Schedule of subjects and investigators

#### Initial visit (baseline)

After receiving informed consent from the patient, the investigator requests a group allocation to the coordinating center. Once the patient has been assigned to a group, a dentist is selected to be in charge. At that time, basic patient information is collected and patient satisfaction and OHIP-EDENT are assessed.

The dentist-in-charge adjusts the jaw-type complete denture in use, if necessary, and schedules the next appointment.

#### Second visit

The investigator evaluates the patient's masticatory performance and food intake. The dentist performs a dynamic impression with tissue conditioner to initiate the intervention.

#### Third visit

The dentist-in-charge places the denture in use once the pain is gone.

#### Fourth visit

The dentist-in-charge places the mandibular CD with a resilient liner in the mouth.

#### A week after the denture is placed

The investigators measures the location and number of mucosal pressure ulcers, patient satisfaction, OHIP-EDENT, and chewing ability.

#### After 3, 6, and 12 months of wearing

The investigators evaluates patient satisfaction, chewing ability, OHIP-EDENT, and food intake.

### Response to adverse events

The investigator, him or herself a dentist, will carefully monitor the occurrence of adverse events. If an adverse event occurs, appropriate measures will be taken and, in principle, the patients will be observed until the event disappears. The type of adverse event, date of occurrence (or date of hospital visit when discovered), details of treatment (continuation, discontinuation, etc.), course of the adverse event, and details of any causal relationship with the resilient liner will be promptly reported to the steering committee. Observations will continue up to 1 year after the application of the resilient liner and will continue throughout the study period.

In this study, there is no anticipated harm and compensation for trial participation.

### Discontinuation of the trial

If any of the following discontinuation criteria are met during the study period, that individual patient will be discontinued promptly at the discretion of the principal/participating investigator: (1) when the subject withdraws consent, (2) when the subject moves to a new hospital due to relocation or stops coming to the hospital for other reasons, (3) when a subject develops symptoms that make it undesirable to start the study, (4) if it is discovered after the start of the study that the subject does not meet the selection criteria or violates the exclusion criteria, and (5) when it is determined that continuation of the study is not desirable for the patient for any other reason.

When the decision is made to terminate the study, the coordinators first report the decision to the steering committee, and if a change in protocol is involved, a breach report is also submitted. The steering committee then notifies the sponsor and the coordinators of each university.

### Data handling

The study coordinators and dentists at each university will identify the patients using registration numbers and initials during the duration of data preparation and handling. They will also take into consideration the protection of patient confidentiality. All of the personnel involved in this study will not be permitted to divulge, without justifiable cause, any confidential information about the individuals involved in the study.

The collected data from each university will be gathered and stored at the coordinating center (Kanagawa Dental University). The chief coordinator will review the collected data, after which the coordinator will perform an interim analysis and then a final analysis. Based on the results of the interim analysis and the status of data accumulation, the coordinating center will decide on the formal termination of the trial.

All materials used in the study will be stored for 3 years after the completion or discontinuation of the study. In principle, access to the final clinical trial data is limited to the coordinator, but any data required to support the protocol can be supplied on request. This trial does not involve collecting biological specimens for storage.

### Statistical analysis

All the analyses will be performed in accordance with the intention-to-treat principle, and various strategies to account for any post-randomization missing data will be contemplated. The statistical analyses will be performed as follows:

1. Normality will be confirmed by the Kolmogorov–Smirnov test, and either parametric or nonparametric tests will be performed.
2. Between-group comparisons will be made using Student’s *t*-test or Mann–Whitney *U* test, depending on the distribution of the outcome.
3. Within-group comparisons will be performed using the paired t-test or Wilcoxon's signed-rank test, depending on the distribution of the outcome.
4. Chi-square test will be used to test proportions.
5. The significance level will be set at 5% on both sides, and p-values will be adjusted by Bonferroni’s correction to account for multiplicity when more than three groups are compared.

Subgroup analyses will also be performed among the different subpopulations defined by each of the multiple baseline characteristics of the participants in order to study whether the effects of the intervention vary across patient characteristics.

## Discussion

The efficacy of single-implant mandibular overdentures to stabilize CDs in edentulous patients has been noted recently [23]. This form of implant therapy is expected to become one of the main treatments for edentulous patients because it involves the insertion of only one implant and thus less surgical invasion and is highly convenient. However, it is difficult to apply this approach in geriatric patients who have systemic diseases or are unable to visit the hospital, and a fundamental solution to this problem has not yet been identified.

In clinical dentistry, the supplementary use of RL materials in the postoperative management of CD wearers has already been recognized [24–26]. Since their introduction in the 1950s, these RL materials have been used to improve the fit of CDs, condition traumatized tissues, and provide an interim or permanent cushioning-like effect. These materials have been reported to help distribute the forces of mastication more evenly and absorb energy [5, 24]. However, most of the previous studies on RL materials have focused on their material properties, including their mechanical properties, the bond strength between the denture resin and the RL, and bonding methods [5]. In contrast, few clinical trials have investigated the clinical effects of RL materials [7–13], and no large clinical study has been conducted to clarify the functionality of RL materials using a multicenter RCT, as in this trial.

In Japan’s rapidly aging society, many elderly CD wearers are unable to visit the dentist's office, despite the fact that poor denture quality can affect their quality of life. The application of RL materials can help some of these patients. However, the lack of sufficient evidence on RL materials has made dentists hesitant to recommend or apply them.

This clinical study has several limitations. First, both the patient and the dentist cannot be blinded to the dental treatment due to its nature. In this study design, it is not possible to conduct a double-blind study. Second, the results obtained have limited generalizability because the sample includes only patients who are able to visit the hospital. Since the external validity of the evidence is lacking as the society further ages, we believe it is necessary to conduct a clinical trial in a new population.

The RCT will provide dentists and prosthetic educators evidence regarding the effectiveness of RL materials for edentulous patients. We believe that the evidence will liberate dentists from the conventional notion that RL materials cause more harm than benefit, and will yield useful information for selecting treatment options for edentulous patients with ill-fitting CDs.

## Trial status and results

The trial study period is January 2021–March 2024 (registration period until September 2023). At the time of manuscript submission (January 26, 2022), patient recruitment is ongoing.

The obtained data will be presented at relevant scientific conferences and submitted to academic journals to help develop diagnostic and therapeutic guidelines for the selection and use of resilient liners.

## Data Availability

The datasets analyzed during the current study and statistical code are available from the corresponding author on reasonable request, as is the full protocol.

## Abbreviations

CD: Complete denture
RL: Resilient liner
NRL: Non-resilient liner
MCORT II: Mandibular Complete dentures with Resilient liner Trial II
RCT: Randomized controlled trial
VAS: Visual analog scale
